# Problems of operation of positive pressure ventilators on the basis of surveys of Polish officers of the State Fire Service

**DOI:** 10.1038/s41598-024-61507-3

**Published:** 2024-05-11

**Authors:** Piotr Kaczmarzyk, Łukasz Warguła, Paweł Janik, Piotr Krawiec, Damian Bąk, Wojciech Klapsa

**Affiliations:** 1 Science and Research Centre for Fire Protection, National Research Institute, 05-420 Józefów, Poland; 2 Institute of Machine Design, Faculty of Mechanical Engineering, Poznań University of Technology, 60-965 Poznań, Poland

**Keywords:** Noise, Exhaust emissions, Occupational safety, Firefighter, Building smoke ventilation, Positive pressure ventilation (ppv), Fire protection units, Survey, Fire ecology, Civil engineering

## Abstract

Positive pressure ventilators (PPV) used by 97.7% of officers of the National Fire Service in Poland, are characterized by work that is not in line with the expectations of the firefighters. In order to improve the technical and operational features of these devices, a survey was conducted among 25,000 eligible firefighters, identifying the application of these devices, problems in use and expected development directions. A total of 682 officers voluntarily completed the survey. Based on their findings, it was determined that ventilators are most often used to smoke out buildings after or during a fire. Mentioned problems when using these devices were mainly noise (78.2%), exhaust emissions (68.5%), and impediments to mobility through the device’s relatively heavy weight (40.2%). Other inconveniences were mentioned by less than 20% of firefighters. Polish firefighters expect the development of these devices mainly in terms of the above-mentioned features (noise reduction (81.7%) and reduction of the weight and size of the ventilators (about 50%)). Other expectations relate to the improvement of smoke removal in buildings: increasing the efficiency of smoke removal (46.4%) and efficiency regarding the rate of smoke removal in a building by increasing the size of the incoming airflow from the building’s surroundings (33.2%). About 15% of firefighters expect changes in the operation of the ventilator itself, that is, an increase in the effective operating time (electric ventilators) and an increase in the device’s uptime. The aim of the article is to identify the issues encountered during the operation and to indicate the expected direction of development for PPV by users. This information can be used by engineers to initiate new development work on these devices.

## Introduction

The activities of the officers of fire protection units are often associated with exposure to various hazards^1–3^. Such risks include exposure to high temperatures^4–6^, smoke emissions (toxic decomposition products)^7–9^ and exposure to strong chemicals^10–12^. In recent years, more and more attention has been paid to aspects of work ergonomics^13,14^ and the physical^15^ and mental health of firefighters^16^. As reported in a statistical study presented by the National Fire Protection Association (NFPA) in 2017, 38% of firefighter deaths in the U.S. were due to cardiovascular problems, 38% to internal injuries, and 15% were related to involvement in an accident^17^. In order to improve the safety and working conditions of firefighters, in addition to statistical studies of accidents, surveys are also carried out to take into account tips to improve work, by the rescuers themselves. Such research can include studies on firefighter ergonomics conducted by Dąbrowska and Bartowiak^18^. The research team presented information on identifying the needs of firefighters in relation to smart protective clothing with a hazard signalling system in surveys. In the survey, respondents showed many important features that this type of clothing should have, including a cooling system, location sensor, thermal imaging camera, and chemical concentration sensor. In addition, the firefighters identified several features related to the ergonomics of this type of clothing, including weight, freedom of movement and durability of the materials. Such research is the basis for determining the direction of development of firefighters’ clothing, e.g., like the aforementioned cooling system^19,20^, location sensor^21–23^, thermal imaging camera^24,25^, chemical concentration sensor^26,27^, weight reduction^28^, improvement of freedom of movement^29^ and material durability^30,31^. Survey tests were also conducted by Roldan-Gomez et al.^32^ on the quality of using firefighters’ equipment. The team studied the tools used in prevention, extinguishment and observation activities in forest areas. In their publication, the authors presented the main problems that accompany rescue operations, such as the lack of human and material resources, lack of sufficient real-time information on the course and development of a fire.

Due to the nature of the firefighter’s work, i.e. working in an extreme fire environment (often under time pressure), in order to minimize the risks they experience they should have the right tools with a high level of reliability. Equipment used to carry out rescue operations is subject to additional testing and certification before it is approved for use. In Poland and other countries, admittance for use of products is determined by the results of laboratory tests, which are carried out for the purpose of carrying out the process of certification and admittance of such products^33^. Unfortunately, not all devices are subject to the admittance process. In case of positive pressure ventilators, no research methods have been implemented to comprehensively evaluate the effectiveness of these devices^34–36^. Ventilators are characterized by a wide spectrum of operation. According to Kaczmarzyk et al.^34^, these devices can be used for, among other things: positive pressure and under pressure ventilation (PPV), positive pressure attack (PPA), assisting evacuation from a fire-affected facility, suppressing fires of free-standing structures, etc.^37^. The group of PPV studies conducted in experimental conditions in unused buildings is being noticed, providing realistic conditions. In such conditions, the effectiveness of firefighting processes and the limitation of negative fire effects are studied, providing guidelines for professional use^38–41^. However, research in such conditions makes it difficult to develop a comparable PPV research methodology.

Taking into account the important role of rescue ventilators in the implementation of rescue operations, CNBOP-PIB (Scientific and Research Centre for Fire Protection—National Research Institute) in Józefów, Poland, has begun to implement the construction of a research infrastructure allowing the evaluation of the most important features determining the effectiveness of this type of equipment. Among the most important characteristics that can determine the effectiveness of rescue operations are: the size of the generated jet; the area of jet dispersion as a function of distance; noise; the length of operating time, as well as the weight and dimensions of the device^42^. The developed range of the apparatus is a flow tunnel made in accordance with the requirements of ANSI/AMCA 240-22 and PN-EN ISO 5801^43,44^. Referring to the importance of the problem related to the design and construction of test apparatus dedicated to the comprehensive conformity assessment processes of all ventilators used in rescue operations, it was also decided to ask firefighters themselves about the aspects of using these devices. As part of this project, it was decided to conduct a survey of officers of the National Fire Service (PSP) in order to most accurately tailor the research program of positive pressure ventilators and specifically address those features that need improvement according to the declarations of direct users—rescue firefighters. The survey were formulated to learn about the frequency of using this type of equipment, to learn about the specifics of using positive pressure ventilators, to identify difficulties and weaknesses related to commissioning and use; and to inquire about technical issues of the ventilators that firefighters believe may need improvement and refinement.

The purpose of the article is to identify operational difficulties and design flaws in emergency ventilators based on the opinions of their users. In addition, the circumstances and frequency of using these devices were determined, along with obtaining information on expected developments. The survey was performed on the basis of questionnaires conducted among professional rescue firefighters directly involved in rescue operations in Poland.

### Ethical issues

The obtained survey results were checked and approved by the Ethics Committee of the Scientific and Research Center for Fire Protection—National Research Institute. The research was conducted taking into account approved guidelines. The consent to conduct the study was given by the Chief Commander of the State Fire Service in Poland. Additionally, each respondent was informed that participation in the study was voluntary. Each respondent who took part in the survey gave informed consent and accepted the terms of participation.

## Material and methods

**Figure 1:**
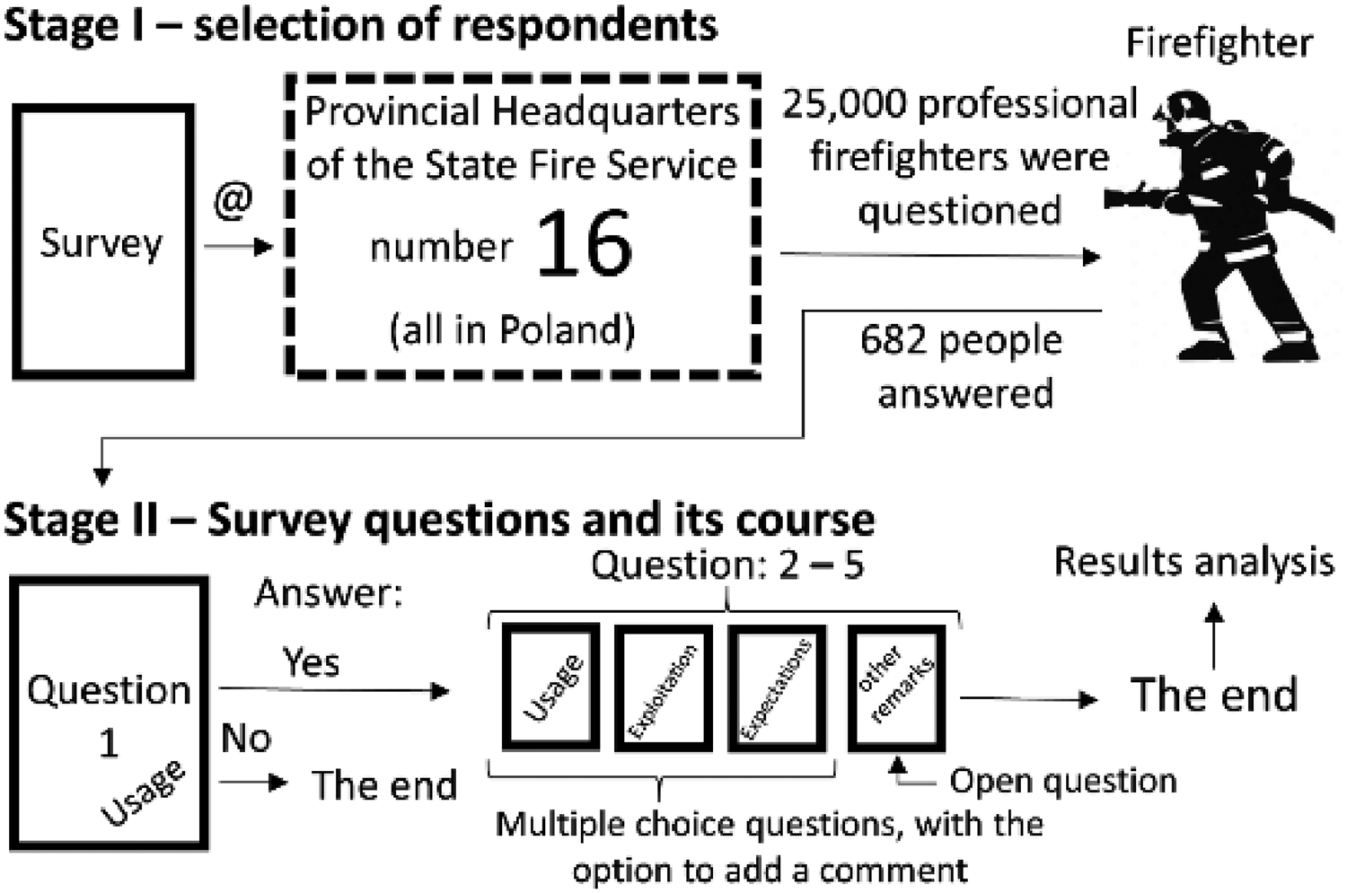
Research plan.

All methods used to conduct the survey were performed in accordance with relevant guidelines and regulations. Aerial surveys were conducted with professional emergency firefighters directly involved in rescue operations employed by the State Fire Service in Poland. The questionnaire form (using the “Google” protocol) was distributed through 16 Provincial Headquarters of the State Fire Service (location: Białystok, Gdańsk, Gorzów Wielkopolski, Katowice, Kielce, Lublin, Łódź, Olsztyn, Opole, Poznań, Rzeszów, Szczecin, Toruń, Wrocław, Warsaw), to 496 subordinate rescue and firefighting units, located throughout the country. The survey was conducted in a voluntary form, so of the 25,000 eligible to participate, 682 officers completed it. The survey was conducted between July 2022 and March 2023 (Fig. 1). It constitutes 2.73% of all professional firefighters in Poland. The first question excluded individuals from the survey who do not use PPV (about 2.3%) during rescue operations, resulting in 666 firefighters responding to questions directly related to working with PPV, representing 2.66% of all professional firefighters in Poland.

The survey contained 5 questions. 4 of the questions were closed-ended, but they also had the option to expand the topic, introduce additional description and expand the answer to the issue the question addressed. Question 5, on the other hand, was strictly open-ended. For the first question only, respondents had a choice of one answer. In other questions, firefighters had the opportunity to select multiple answers.

The first question (1) addressed the issue of using mobile ventilators “Do you use mobile ventilators during rescue operations or cyclic exercises?”. The indicated question was in a way identifying the respondent. The respondent had the option to answer the question in two ways, i.e. yes or no. If the answer was affirmative to the question, the respondent moved on to the next question in the survey. On the other hand, if he or she denied it and selected the answer ‘no’, at this stage this resulted in terminating participation in the survey.

The second question (2) was related to identifying the activities for which firefighters use positive pressure ventilators. The question was “What activities do you use the positive pressure ventilator for?”. In the area of closed questions, the respondent had a choice of 6 answers, labelled a to f respectively, which were:

- Removal of thermal decomposition products and accumulated heat in the building space (technique: positive pressure ventilation);
- Removal of thermal decomposition products and accumulated heat in the building space (technique: vacuum ventilation);
- Smoke removal from the building space after the location of a fire (smoke extraction);
- PPA positive pressure attack (combination of extinguishing action and mechanical ventilation with mechanical positive pressure ventilation);
- Action on fires in free-standing structures (e.g. cars, waste containers);
- Providing air to areas where people may be present and oxygen concentrations may be reduced.

For the indicated question, respondents were also given the opportunity to include an additional open-ended comment, where they could indicate additional activities for which positive pressure ventilators are used.

The next question (3) in the survey concerned the difficulties associated with the use of positive pressure ventilators. Respondents read the question “What difficulties did you encounter during the activities of making and using a positive pressure ventilator?” As part of the closed answers assigned to the indicated question, firefighters were given the opportunity to select 15 answers (letter designation ‘a’ to ‘o’), which related to the following operational problems:

- Insufficient efficiency of the incoming airflow generated by the ventilator;
- Ventilator operating time;
- Too large a unit;
- Impaired mobility of the unit (e.g. too much weight);
- Presence of defects related to ventilator mechanisms (e.g. changes in the discharge angle);
- Malfunctions in the drive system;
- Defects in ventilator components related to poor workmanship;
- No markings indicating the optimal position of the unit;
- Problems with the power supply;
- Noise;
- Exhaust gas emissions (for ventilators driven by an internal combustion engine);
- Inability to provide a proper inlet or inlet opening;
- Inability to provide an effective gas exchange path (supply-exhaust distance);
- Problems with the correct positioning in terms of distance and angle of discharge in front of the inlet or outlet opening;
- Adverse environmental conditions affecting rescue operations (e.g. strong wind).

For this question (3), firefighters were also given the opportunity to include an additional response within an open-ended description, where they could indicate difficulties and problems associated with the operation of positive pressure ventilators other than those listed.

Question four (4) of the survey was aimed at identifying the weaknesses of positive pressure ventilators and identifying directions for improvement of the equipment. The question was as follows: “What are your expectations for improving the technical and performance characteristics of positive pressure ventilators? Which features should be modified?”. In this case, respondents were able to mark up to seven responses at a time, indicated by the letters ‘a’ to ‘g’. The closed-form answers were as follows:

- Increasing in the inflow volume of the airflow generated by the mobile ventilator (capacity);
- Extending of the effective operating time of the mobile ventilator;
- Reducing the size of the ventilator;
- Reducing the weight of the unit;
- Reliability (failure-free);
- Reducing noise levels;
- Increasing the efficiency of flue gas extraction.

In this case, respondents also had the option to include an additional open-ended response.

The last question (5) included in the survey was typically open-ended and its purpose was to identify additional insights related to the operation of positive pressure ventilators not previously asked about in the survey. The question was: “What are your other observations that you would like to share regarding the technical and operational performance of positive pressure ventilators?”. As part of this question, respondents were given the opportunity to formulate any observation about the equipment and to point out various problems related to its operation.

The statistical analysis of the survey results involved calculating the cumulative value of the normal distribution function and determining the significance level of the obtained results based on this. Subsequently, the statistical significance of selected responses chosen by respondents was assessed relative to the null hypothesis, which assumed that each response had a significance level of p = 0.5. This means that in the null hypothesis, at least half of the respondents had to choose each of the responses in the questions asked. Based on this, it was demonstrated which technical aspects are significant for further construction processes, prioritizing them accordingly.

## Results and discussion

Analysing the answers to the first question on using mobile ventilators during rescue operations or cyclic exercises, out of all the respondents (682 firefighters), 666 replied in the affirmative, i.e. 97.7% of the respondents. In contrast, a negative response was indicated by 2.3% of respondents (16 people). According to Lambert and Merci, PPV has been widely used among Belgian firefighters for more than 20 years^45^. The use of PPV significantly improves the safety of firefighters and rescued persons hence it is widely used as clearly confirmed by the scientific community^46–48^.

**Figure 2:**
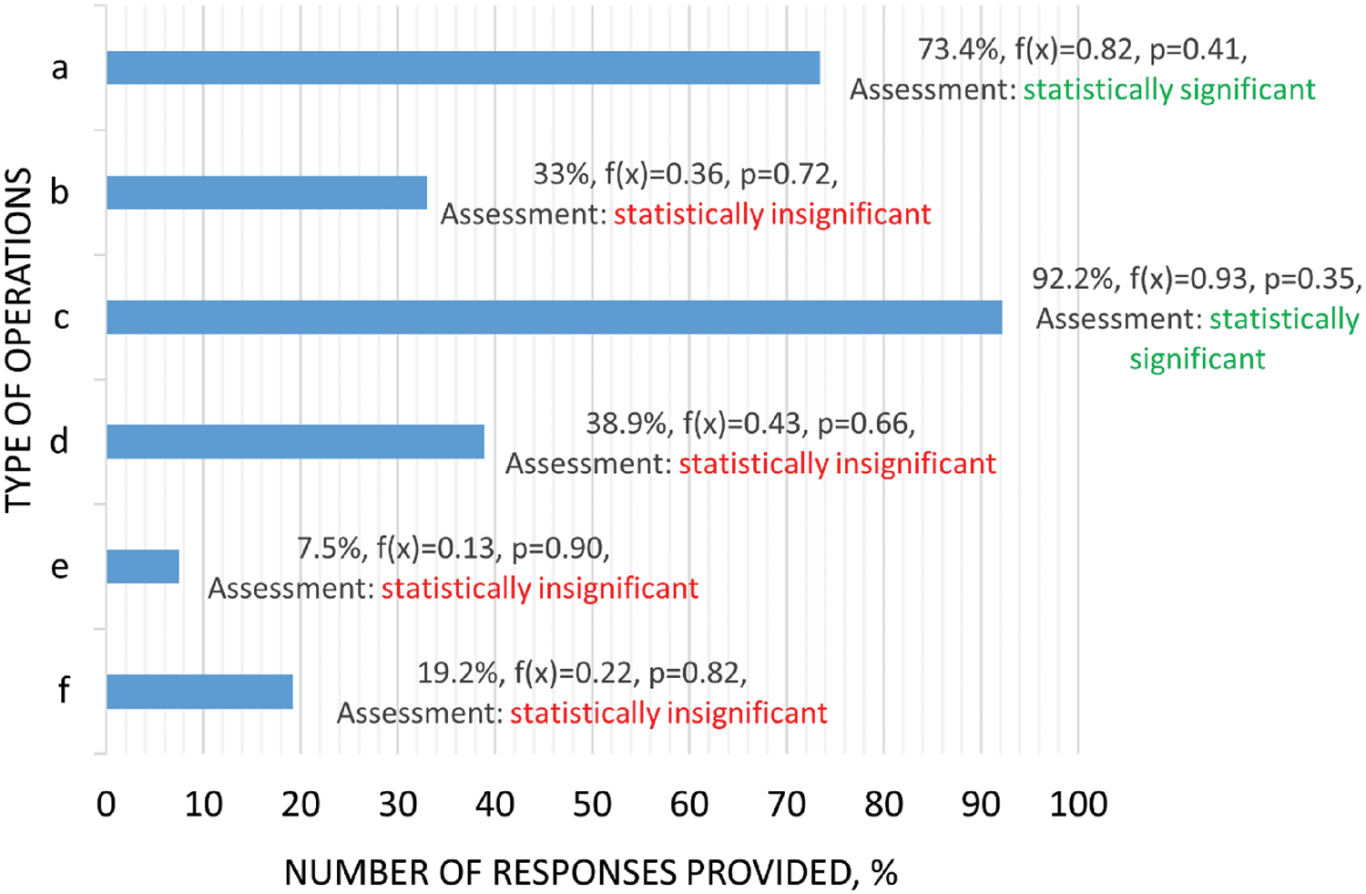
Activities for which firefighters use positive pressure ventilators, where: a—removing thermal decomposition products and accumulated heat in the building space (technique: positive pressure ventilation); b—removing thermal decomposition products and accumulated heat in the building space (technique: negative pressure ventilation); c—removing smoke from the building space after the fire has been located (smoke extraction); d—PPA positive pressure attack (combination of extinguishing action and mechanical positive pressure ventilation); e—operations involving fires on free-standing objects (e.g. cars, waste containers); f—supplying air to areas where people may be present and oxygen concentrations may be reduced, f(x)—normal distribution, where x—is a random variable, i.e. the number of answers given to the question, p—level of statistical significance, descriptive assessment based on the stated null hypothesis.

Referring to the results of the second question related to the identification of activities in which positive pressure ventilators are used, respondents emphatically showed that the most common activity is the removal of smoke from a building space after a fire has been extinguished (92.2% of respondents). As the second most frequently mentioned activity, firefighters indicated the implementation of smoke extraction during internal fires using positive pressure ventilation (72.4% of respondents). Responses related to smoke extraction are the most anticipated, as a large group of researchers also raise the issue for this purpose^49–51^. The least popular answer among respondents was ‘e’, i.e. Actions related to fires of detached structures (7.5%) (Fig. 2). The issue of extinguishing, for example, cars^52^ using PPV is a little recognised scientific topic and may also be little publicised among professional firefighters. As part of their open-ended response to this question, firefighters listed situations in which they use PPV:

- supplying air for faster burning of hydrophobic material;
- generation of positive pressure in passageways to ensure evacuation conditions;
- supplying more air to a fire in order to burn it out more quickly (e.g. haystacks);
- cooling of objects heated by high temperatures;
- ventilation of the facility after a gas leak using an explosion-proof ventilator to reduce its concentration;
- removing carbon monoxide from dwellings;
- routing of fire gases.

The information obtained from the open-ended responses is often a new or non-classical approach in the application of PPV during rescue and firefighting operations. Using PPV to post-combust hydrophobic material is not an encountered approach in the literature and requires research into the justification of this method. However, the issue of using hydrophobic materials in extinguishing processes is a current scientific topic, e.g. hydrophobic coatings in dry water methods^53^, extinguishing gel^54^, hydrophobic silica nanoparticles as additives to improve extinguishing agents^55^. Methods of controlled afterburning of ignitable material are justified and, as Kerber and Walton in 2005 point out, PPV can contribute to an increase in the rate of combustion^56^. The delivery of an air jet can facilitate the combustion of compacted materials^57–59^. The routing of exhaust gases and the cleaning of passageways using PPVs are extinguishing methods known and described, for example, by Alianto et al.^60^. In contrast, the cooling of objects heated by high temperatures is described by Madrzykowski and Kerber^61^ and Giełżecki and Wolański of the protective clothing of firefighters^62^.

**Figure 3:**
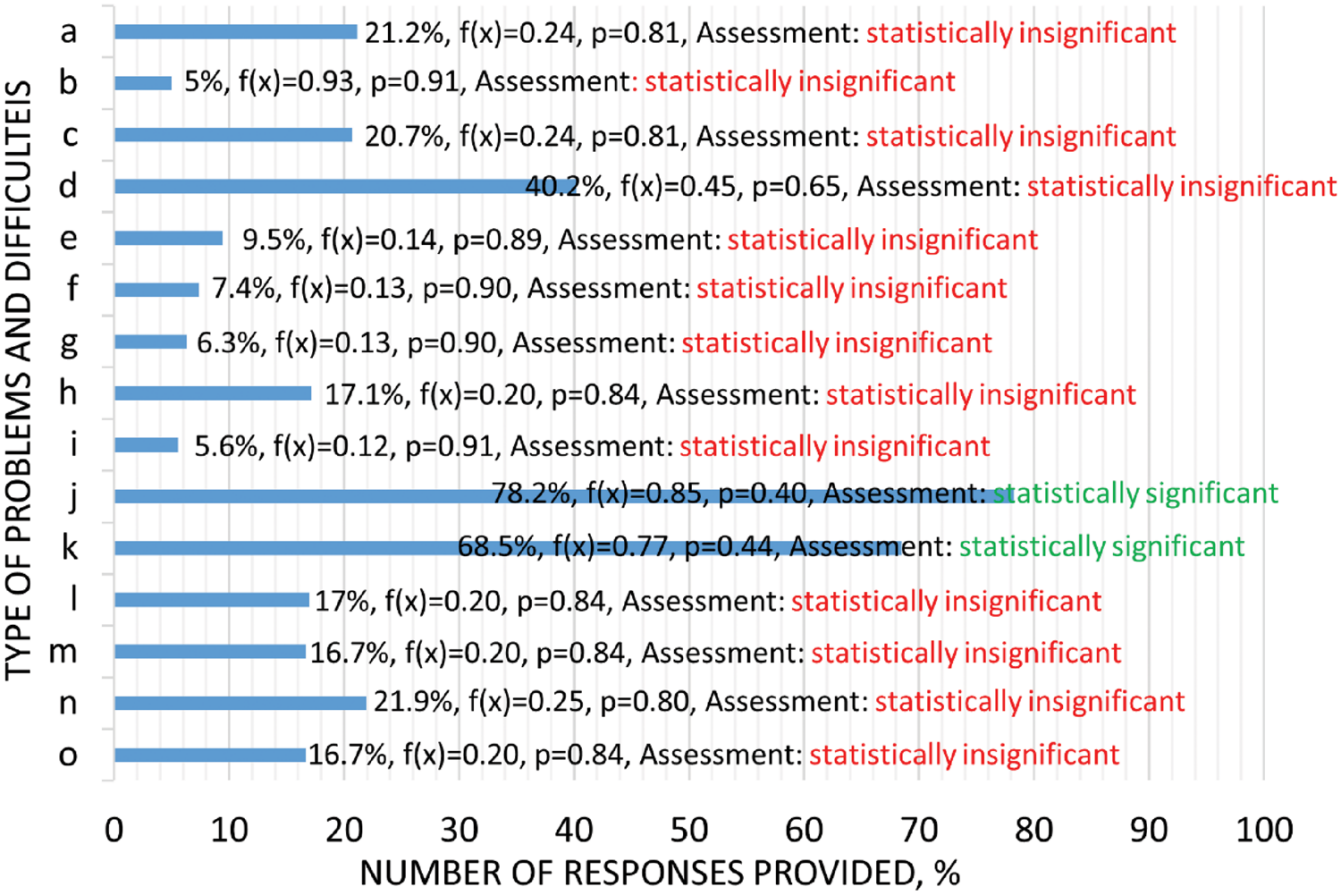
Difficulties encountered during ventilator set-up and operation, where: a—insufficient inflow capacity of the airflow generated by the ventilator; b—operation time of the ventilator; c—unit size too large; d—impaired mobility of the unit (e.g. too much weight); e—presence of defects relating to ventilator mechanisms (e.g. changes in the discharge angle); f—defects in the drive system; g—defects in ventilator components related to poor workmanship; h—no markings indicating the optimal position of the unit; i—problems with the power supply; j—noise; k—exhaust gas emissions (for ventilators driven by an internal combustion engine); l—inability to provide a proper inlet or inlet opening; m—inability to provide an effective gas exchange path (supply-exhaust distance); n—problems with the correct positioning in terms of distance and angle of discharge in front of the inlet or outlet opening; o—adverse environmental conditions affecting rescue operations (e.g. strong wind), f(x)—normal distribution, where x—is a random variable, i.e. the number of answers given to the question, p—level of statistical significance, descriptive assessment based on the stated null hypothesis.

In question three, respondents answered a question about the type of difficulties encountered during the activities of setting up and using the ventilators. Firefighters demonstrated a number of problems (Fig. 3). The biggest problem identified by respondents is the accompanying noise (78.2%) and emissions of harmful exhaust gases, with equipment powered by combustion engines (68.5%) and difficulties in the mobility of the units, e.g. high weight (40.2%). Other inconveniences with using ventilators are indicated by between 21.9% and 5% of firefighters.

Analysing the identified problems, future research should be carried out into what is the main source of noise: the operation of the combustion engine or the ventilator impeller. Development work is being carried out in both groups of noise sources. Noise-reducing rotor and stator geometries are being developed among ventilator designs^63,64^. On the other hand, in the midst of internal combustion engines, vibration and noise reduction is attempted by improving the combustion of the fuel–air mixture^65,66^, the design of mechanisms such as injection valves^67^ or improving the damping of exhaust systems^68^. In the small-engine non-road designs used to drive rescue ventilators, the exhaust systems (which also dampen noise) through the compact design are quite small^69,70^. One way to reduce the negative impacts (noise^71^, emissions of harmful exhaust gases^35,72,73^, high temperatures^69,70^) of internal combustion engines is to replace them with electric motors^74,75^. However, within the group of emergency ventilator drive units, as demonstrated by Warguła and Kaczmarzyk^36^ on the Polish market the most popular are combustion powered ventilators with a power output ranging from 3.4 to 6.3 kW (approximately 50% of the market). On the other hand, electric ventilators are commonly offered in the smaller power range from 1.1 to 2.2 kW (74% of the electric ventilator market in Poland)^36^. Hence, for the time being, replacing internal combustion drives with electric drives, especially of greater power, seems difficult to implement in practice.

The impediments to mobility when using these units, resulting from the high weight, can be reduced by using lighter construction materials for frames^76,77^, ventilator impellers^78^ and motors^79,80^. Such materials are known, but the current problem is to produce ventilator parts from lightweight materials at a competitive price.

**Figure 4:**
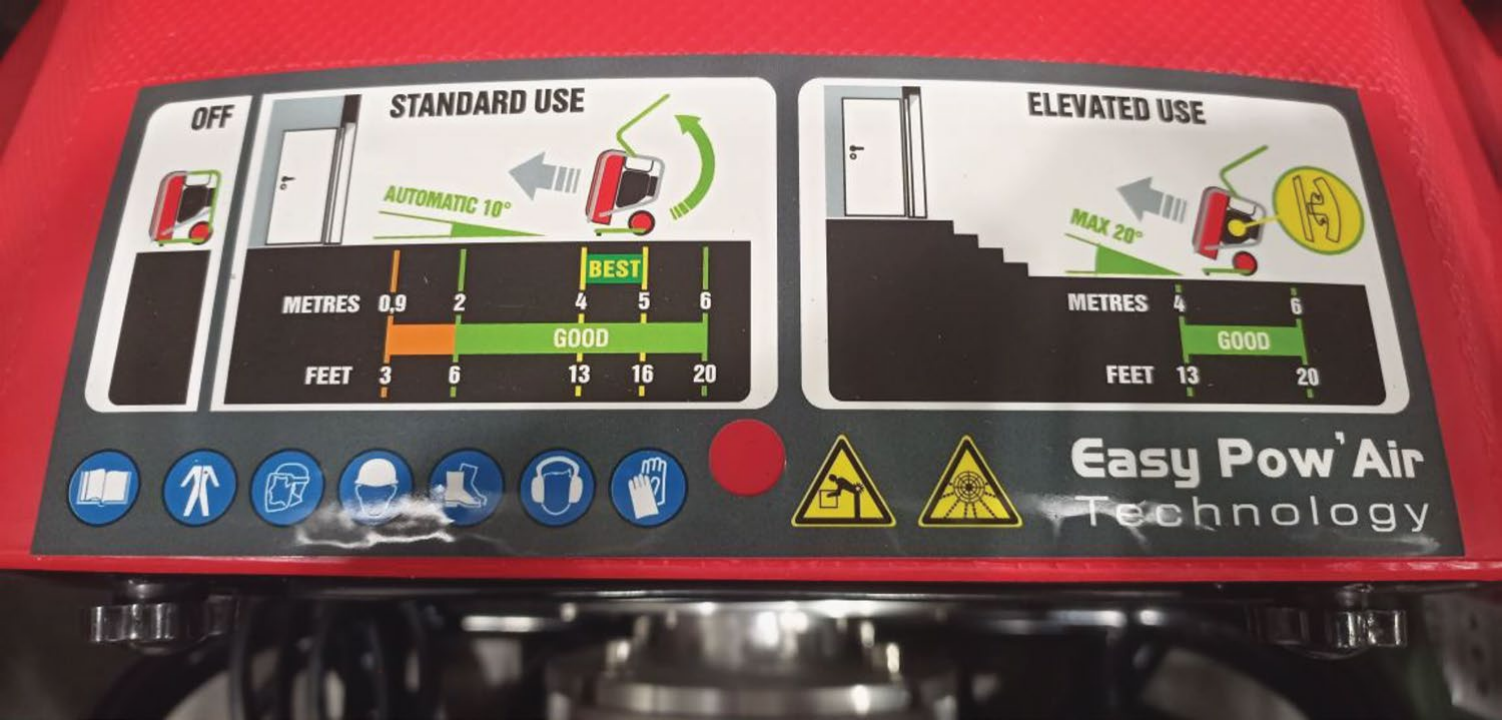
Information to assist in positioning the rescue ventilator.

Problems in using emergency ventilators exhibited by a group of about 10–20% of users include problems with the correct positioning of the unit: lack of markings indicating the optimal positioning of the unit, problems with the correct positioning in terms of distance and angle of the discharge in front of the inlet or outlet opening. Some manufacturers introduce markings attached to the ventilators to aid their correct positioning (Fig. 4). Measurement systems to aid their positioning could be one way forward, but there are none at present. Another perceived problem is the inadequate inflow capacity of the airflow generated by the ventilator, which can be improved by using more powerful drive units. At the same time, increasing the efficiency of the drive units can solve another problem: the unit is too large. Another group of problems relates to architectural considerations related to the inability to provide a proper inlet and intake opening, or the inability to provide an effective gas exchange path (supply–exhaust distance). Currently, this problem is in the minds of architects and they are taking such problems into account. For many structures, analyses of smoke movement are carried out in a computational fluid dynamics (CFD) environment^81–83^ to determine the precise geometry of buildings or tunnels, for example. The last problem mentioned in this group was adverse environmental conditions affecting rescue operations (e.g. strong winds). Wegrzynski and Krajewski showed that wind force and the location of smoke extraction ventilators (roof or wall-mounted) significantly affect the smoke extraction capacity of buildings (up to 37%)^84^. Research is being conducted into the correlation of wind force and direction on a building’s smoke^85^ or fire spreading capacity^86^ that seeks to improve firefighting methods. However, there are no clear guidelines for the positioning of rescue ventilators in such a situation.

**Figure 5:**
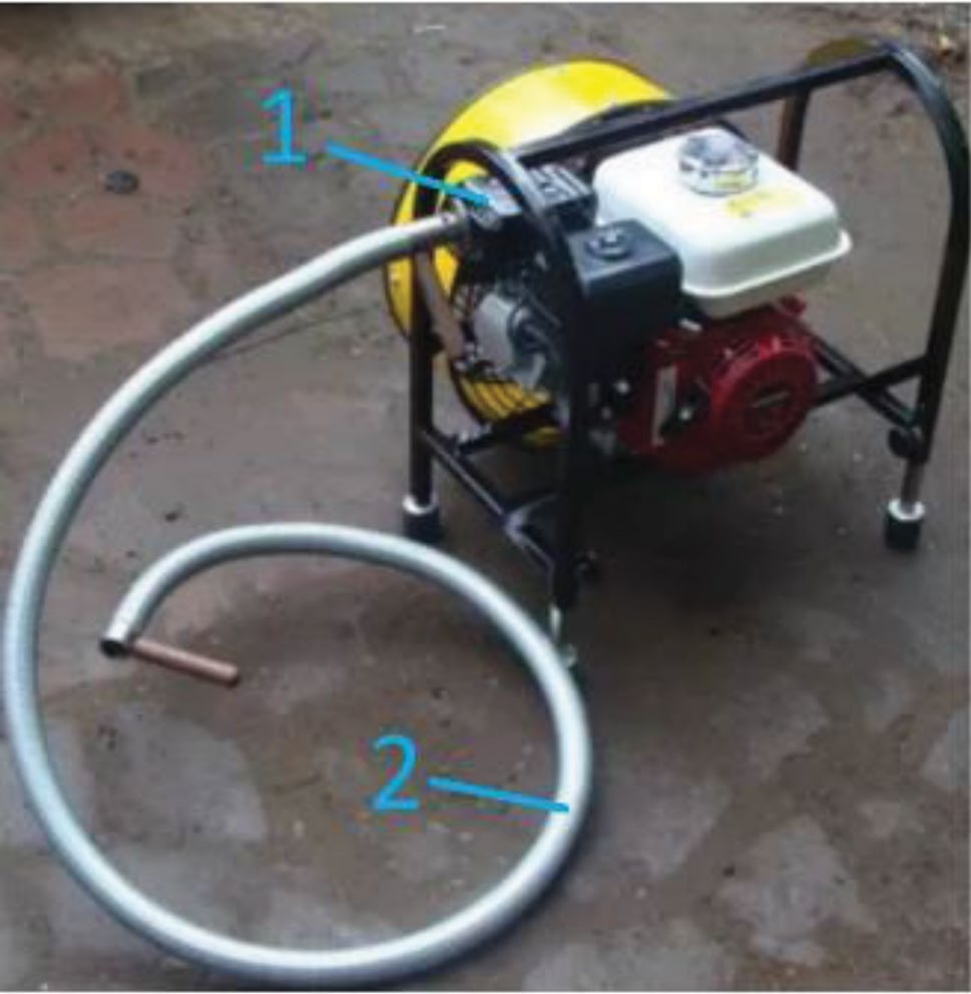
Rescue ventilator with exhaust system from the combustion engine, where: 1—exhaust manifold with silencer, 2—exhaust gas duct.

The problems perceived by a group of less than 10% of firefighters using ventilators are those related to the ventilator drive unit, i.e. its faults and operating time. According to the survey, the drive units used in the ventilators are of relatively high quality, with only 7.4 per cent of respondents reporting problems, although it is possible that some of them are using equipment that has been in operation for a long time and may have malfunctions due to wear. With regard to operating time, internal combustion engines are characterised by the absence of operating time limitations when refuelling, whereas battery-driven ventilators in this situation require the replacement of batteries or batteries with increased energy capacity. 9.5% of respondents perceive problems with mechanisms, e.g. changing the angle of the ventilators, and 6.3% other faults related to the poor quality of manufacture of these devices. The solution to this problem may lie in control tests that determine whether products are approved for use by the fire service. Additional problems reported by respondents include the injection of combustion products generated by the ventilators’ combustion engine into the smoke-filled building (they are sucked up by the jet and injected into the building). These gases are also detected by carbon monoxide detectors, distorting the reading. The solution to this problem lies in systems that extract the exhaust gases outside the ventilator’s air intake area (Fig. 5). In addition to exhaust emissions, a perceived problem is the vibration of the diesel-powered device, which can result in a change in its position, relative to the initial setting. This problem was studied and analysed by Wieczorek et al.^48^ indicating values for the selection of vibration dampers and vibration isolators that can prevent the equipment from affecting the ground or the operators. Another problem relates to the positioning in the fire vehicle, where it is, according to respondents, positioned too high. This is an individual problem for manufacturers of firefighting vehicle equipment bodies, but this information can be a new design guideline, as the main emphasis in the design methodology to date has been on the position of the vehicle’s centre of gravity^87^.

**Figure 6:**
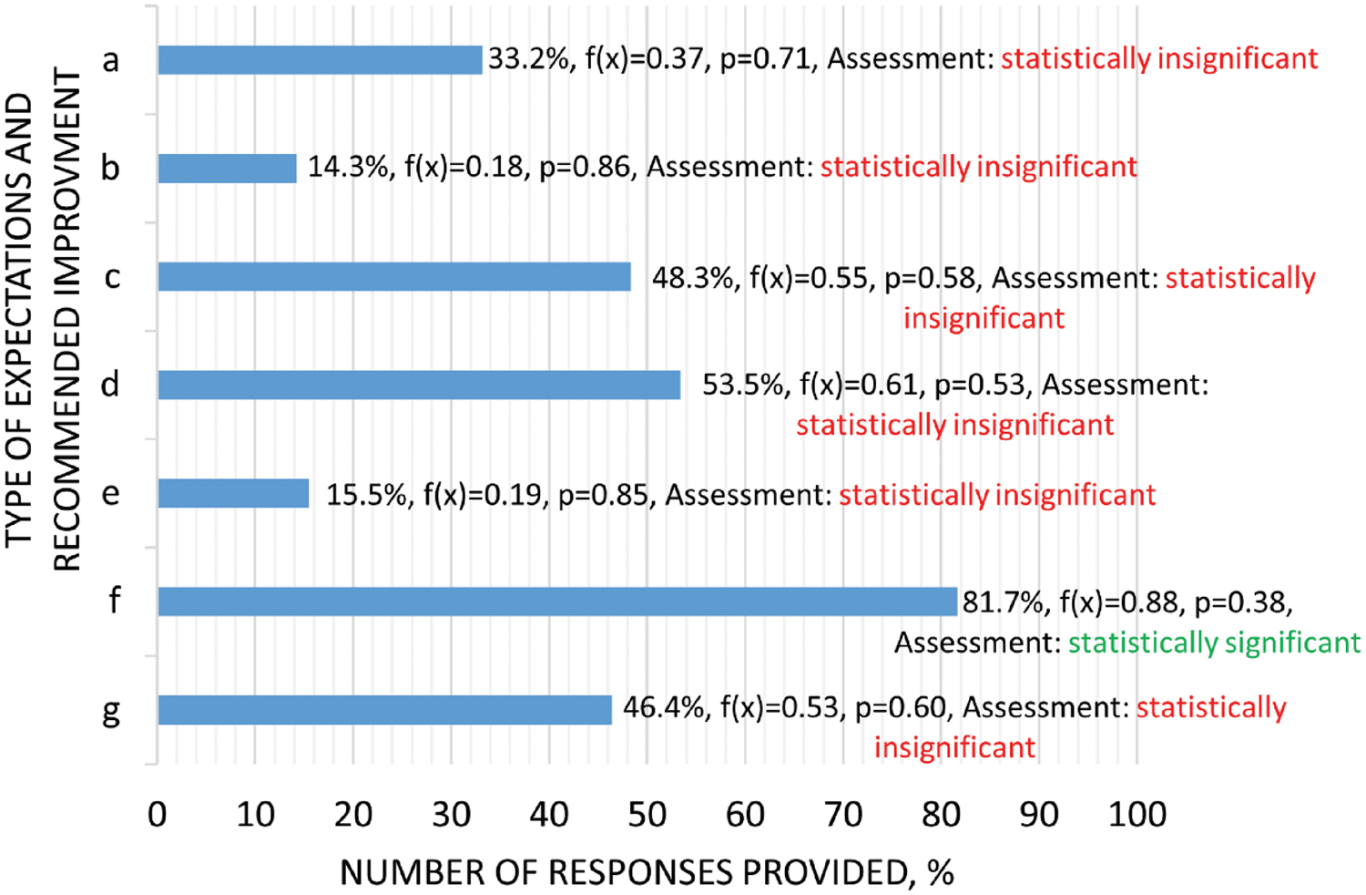
Expectations of firefighters towards the development of rescue ventilators, where: a—increasing in the inflow volume of the airflow generated by the mobile ventilator (capacity); b—extending of the effective operating time of the mobile ventilator; c—reducing the size of the ventilator; d—reducing the weight of the unit; e—reliability (failure-free); f—reducing noise levels; g—increasing the efficiency of flue gas extraction, , f(x)—normal distribution, where x—is a random variable, i.e. the number of answers given to the question, p—level of statistical significance, descriptive assessment based on the stated null hypothesis.

Question four focused on firefighters’ expectations and indicated the expected developments for mobile ventilators based on user feedback. The largest percentage of users, 81.7%, expect a reduction in noise levels and up to 53.5% to 48.3% expect a reduction in weight and size. This is the most popular expectation among firefighters, and it is noticeable that these are issues that directly affect them when transporting the equipment or operating it. The next comments relate to improvements in the extraction capacity of smoke from buildings: 46.4 per cent of respondents expect to increase the efficiency of smoke extraction and 33.2 per cent expect to improve the building’s air purification capacity by increasing the size of the incoming airflow from around the building. Between 14.3 and 15.5% of respondents expect an increase in the effective operating time of the mobile ventilator and an increase in the device’s reliability (Fig. 6).

Additional expectations and desired improvements reported by respondents include, for example, increasing the range of possibilities for setting the angle of the ventilator impeller (also introducing control of the direction of the airstream below the horizontal axis). Currently, in the commonly used units, the value for changing the position of the ventilator impeller from the horizontal is between 0° and 18° (upward airflow direction), with the most common four recommended positions^88^. The remaining open-ended responses related to drive units, reducing CO emissions from internal combustion engines and reducing the cost of purchasing electric-powered ventilators with increased uptake.

The last question was open-ended and concerned proposals for improving the technical and performance characteristics of mobile ventilators. Only those not mentioned directly or indirectly in the earlier questions were analysed. The development or dissemination of dedicated extractor ventilators is expected. Such a solution could be useful for smoke extraction in confined spaces where the positive pressure method is not effectively applicable (e.g. basements without windows). With regard to this aspect, aeration sleeves are a helpful tool. With this solution, it is possible to supply air to any area of the room to be ventilated and the inlet opening (e.g. a door) can be used as an exhaust point. An important feature advocated by respondents is the design of ventilator housings to facilitate cleaning. Such structures are contaminated with soot and oily carbon build-up especially on the blades and rotor housing and cowl after smoke removal. Currently, most commercial designs are characterised by the time-consuming and complicated removal of the rotor housing, which makes maintenance of the unit difficult. At the same time, problems are reported with the installation of the light foam net or the compatibility of the aeration sleeves. Firefighters expect the aeration sleeves to be standardised, as they see problems connecting them and connecting them to ventilators if they are made by different manufacturers. Ventilator operators also expect wheels to aid their mobility, as not all units are equipped with them, and wheel chocks for units with significant vibration. Limitations on the use of positive pressure ventilation due to the extinguishing of the internal combustion engine due to a deficit of oxygen in the fuel–air mixture. The advantages and disadvantages of electric and combustion fan drives are recognized, hence there are proposals for electric-fuel hybrid drives. We can conclude that the authors have in mind a kind of parallel hybrid, but a solution where two sources of propulsion would work simultaneously would not eliminate most of the disadvantages. Thus, it can be concluded that the authors of the answer are referring to a fan with two independent drives, used depending on demand and operating conditions, for example, in an open space with a combustion drive, and in a closed space with an electric drive. A similar increase in the universality of the fan can be achieved by a hybrid series drive, which would rely mainly on a fan with an electric motor powered by a generator through electric wires from a greater distance, the solution eliminates one of the major drawbacks of electric fans, namely the battery (the main disadvantages cited: limitation in energy storage capacity, durability, purchase cost).

In addition to design expectations, firefighters report a need for more training in the area of using ventilators during rescue operations, especially with regard to operations during a fire. Firefighters also expect inspections of the equipment admitted for use, as there are known cases of new equipment being purchased with very low efficiency, and thus ineffective in rescue operations. The number of ventilators per unit in a particular province is also expected to increase.

## Conclusion

One of the ways to identify directions for the development of mobile ventilators, in terms of improving their use, are surveys of firefighters who actively use these devices during rescue and firefighting operations. Firefighters in Poland use these devices 92.2% of the time to ventilate buildings after a fire is extinguished or during a fire (72.4%). One of the non-classical uses of these devices is to supply more air to a fire in order to burn it out faster (e.g. haystacks). Difficulties cited during the use of these devices were mainly: noise (78.2%), exhaust emissions (68.5%), and difficulties in mobility through the relatively heavy weight of the device (40.2%). Other inconveniences were mentioned by less than 20% of firefighters. Polish firefighters expect the development of these devices in terms of direct impacts on their bodies, namely reducing noise (81.7%) and decreasing the weight and size of fans (about 50%), which require manual transport. Other expectations include improvements in building smoke removal: increasing the efficiency of smoke removal (46.4%) and the efficiency of building air purification by increasing the volume of incoming airflow from the building’s surroundings (33.2%). About 15% of firefighters expect changes in the operation of the ventilator itself, that is, an increase in the effective operating time (electric fans) and an increase in the unit’s uptime. Among the firefighters’ comments in terms of improving technical and operational performance, not mentioned earlier, they expect: efficient and commercially available exhaust ventilator designs dedicated to smoke removal in confined spaces, ventilator housing designs that facilitate cleaning, easy-to-install and inter-manufacturer-compatible accessories such as aeration sleeves or foam systems, and wheeled transport support systems. In addition to the design expectations of firefighters, they report the need for more training in the area of ventilator use during rescue operations, especially taking into account actions during a fire. Firefighters also expect to inspect the equipment admitted for use, as there are known cases of new equipment being purchased with very low efficiency, and thus ineffective in rescue operations. In the existing literature, there is no analysis of expectations in the development direction of mobile ventilators, which studies have shown should continue to be developed structurally and their users trained in their use. A limitation of the research is the problem of using mobile ventilators by professional firefighters in the territory of only one of the European Union countries. These problems can vary significantly in more or less developed countries. Further research should be conducted on design changes to these units, e.g. less impactful drive units, more efficient air jet generators, more ergonomic frames during transport.

## Data Availability

The datasets generated and analysed during the current study are available in the Google Cloud repository: https://drive.google.com/drive/folders/1Qhhm8iD36sE9GLYU2hJhGBzzPot6ZhSP?usp=sharing.
